# Cytokeratin 8/18 expression indicates a poor prognosis in squamous cell carcinomas of the oral cavity

**DOI:** 10.1186/1471-2407-6-10

**Published:** 2006-01-13

**Authors:** Thomas Fillies, Richard Werkmeister, Jens Packeisen, Burkhard Brandt, Philippe Morin, Dieter Weingart, Ulrich Joos, Horst Buerger

**Affiliations:** 1 Department of Cranio-Maxillofacial Surgery, University of Muenster, Waldeyerstrasse 30, 48129 Muenster, Germany; 2 Department of Oral and Maxillofacial Surgery, Central German Armed Forces Hospital, Rübenacher Str. 170, 56072 Koblenz, Germany; 3 Institute of Pathology, Klinikum Osnabrueck, Germany; 4 Institute of Tumor Biology, Center of Experimental Medicine, University Medical Center Hamburg-Eppendorf, Martinistr. 52, 20146 Hamburg, Germany; 5 Department of Maxillofacial Surgery, Katharinenhospital Stuttgart, Kriegsbergstrasse 60, 70174 Stuttgart, Germany; 6 Institute of Pathology, University of Muenster, Domagkstraβe 17, 48149 Muenster, Germany

## Abstract

**Background:**

Intermediary filaments are involved in cell motility and cancer progression. In a variety of organs, the expression of distinct intermediary filaments are associated with patient prognosis. In this study, we seeked to define the prognostic potential of cytokeratin and vimentin expression patterns in squamous cell carcinomas (SCC's) of the oral cavity.

**Methods:**

308 patients with histologically proven and surgically treated squamous cell carcinomas of the oral cavity were investigated for the immunohistochemical expression of a variety of intermediary filaments including high- and low-molecular weight cytokeratins (Ck's), such as Ck 5/6, Ck 8/18, Ck 1, CK 10, Ck 14, Ck 19 and vimentin, using the tissue microarray technique. Correlations between clinical features and the expression of Cytokeratins and vimentin were evaluated statistically by Kaplan-Meier curves and multivariate Cox regression analysis.

**Results:**

The expression of Ck 8/18 and Ck 19 were overall significantly correlated with a poor clinical prognosis (Ck 8/18 p = 0.04; Ck19 p < 0.01). These findings could also be reproduced for Ck 8/18 in primary nodal-negative SCC's and held true in multivariate-analysis. No significant correlation with patient prognosis could be found for the expression of the other cytokeratins and for vimentin.

**Conclusion:**

The expression of Ck 8/18 in SCC's of the oral cavity is an independent prognostic marker and indicates a decreased overall and progression free survival. These results provide an extended knowledge about the role of intermediary filament expression patterns in SCC's.

## Background

Intermediary filaments, like cytokeratins are essential intracellular components, underlying or reflecting distinct cellular properties and differentiation stages in epithelial organs. The proteins of the cytokeratin family are epithelium specific expressed [1] as low and high-molecular weight, resp. acid and basic polypeptides. Squamous, stratified epithelium is usually characterized by the expression of CK 5, which is found mainly in the basal cell layers and Ck 5 is associated with the proliferative potential of these cells [2,3]. The intermediary cell layers show an additional expression of Ck's 1 and 10, which are regarded as signs of cellular differentiation [2]. In contrast, glandular epithelia reveal the expression of low-molecular weight cytokeratins Ck's 8/18 and 19 as typical features – expression pattern of Ck 8/18 is rather uncommon in mature squamous epithelium. Ck 19 expressed heterogeneously in the basal cell layers of stratified squamous epithelium [2,3]. Suprabasal expression of Ck 19 seems to be correlated with premalignant transformation in oral epithelium [4]. The expression of vimentin is mainly regarded as sign of a mesenchymal differentiation. However, vimentin-positively has repeatedly reported in various carcinomas and was interpreted as sign of an epithelial-mesenchymal transition, indicating an increased metastatic potential [5-9].

Recently, it could be shown that the induction of Ck 8/18 expression in non-malignant buccal mucosa cells resulted in a significant change of phenotypic characteristics after Ck 8/18 transfection [10]. These changes included an increased cellular motility, which might give first hints for an increased tumour aggressiveness and poor patient prognosis. However, these findings have not been transferred on a clinical level, yet. Therefore, the significance of these findings in a clinical context, especially in regard to the prognostic importance is unclear.

It was therefore the aim of this study to evaluate the expression of intermediary filaments in SCC's in relation to clinico-pathological features, especially for prognostic purposes by the use of tissue micro arrays and immunohistochemistry.

## Methods

Patients with histologically proven squamous cell carcinoma of the oral floor treated surgically were eligible for the study. All patients underwent surgical treatment including radical resection of the whole tumour with a free histopathological margin of at least 4 mm from the tumour borders. Selective neck dissection of Level I, II, III and V was performed in case of suspect results in preoperative tumourstaging by computertomography or sonography examination or in case of tumour size over 2 cm, bilateral selective neck dissection was performed when the tumour spread the midline (according to the recommendation of Robins et al. 2002 [11]). Radiotherapy was performed when lymph node metastases were detected histologically. All tumours were classified postsurgically according to the TNM system 6^th ^eds. UICC 2002 [12].

## Method

Tumour specimens of 308 patients were investigated for the expression of Ck 5/6, Ck 8/18, Ck 1, CK 10, Ck 14, Ck 19 and vimentin (Table 1) by means of the tissue micro array (TMA) technique.

For tissue micro array (TMA) construction, two punch biopsies with a diameter of 0.6 mm from each donor block were taken and transferred into the new acceptor block. TMA construction was performed by using a special tissue micro array instrument (Beecher Instruments, New Jersey, USA), according to standard protocols [13,14].

Immunohistochemistry was performed on 4-μm-thick TMA sections. After deparaffinization and rehydration, endogenous peroxidase activity was blocked for 30 minutes in methanol containing 0.3% hydrogen peroxide. After antigen retrieval, a cooling-off period of 20 minutes preceded the incubation of the primary antibody. Thereafter, the catalyzed signal amplification system (DAKO, Glostrup, Denmark) was used for Ck 5/6, Ck 8/18, Ck 1, CK 10, Ck 14, Ck 19 and vimentin staining according to the manufacturer's instructions.

All antibodies were detected by a standard avidin-biotin complex method with a biotinylated rabbit anti-mouse antibody (DAKO) and an avidin-biotin complex (DAKO). The stainings were developed with diaminobenzidine or alternatively using LSAB/AP (DAKO). Before the slides were mounted, all sections were counterstained for 45 seconds with hematoxylin and dehydrated in alcohol and xylene. Appropriate negative controls (obtained by omission of the primary antibody) and positive controls were used throughout.

The expression of Ck 5/6, Ck 8/18, Ck 1, CK 10, Ck 14, Ck 19 and vimentin were determined independently by two pathologists (HB and JP). Both pathologists determined the percentage of positive cells in each core.

The percentage was classified for Cytokeratin 19 in 3 groups (0 % no Expression, 1:1–50% moderate expression, 2 >50% high expression) and Ck 5/6, Ck 8/18, Ck 1, CK 10, Ck 14 and vimentin in two groups (0: no Expression, 1: ≥1% positive expression). The mean percentage value of the two cores representing one tumour and were used for further evaluation.

### Statistical analysis

The TNM-stage, the histological differentiation and the expression of Ck 5/6, Ck 8/18, Ck 1, CK 10, Ck 14, Ck 19 and vimentin were related to the duration of the progression-free and the overall survival. The measurement of time started from the date of surgery to the date of histologically proven recurrent or metastatic carcinoma or disease related death, respectively. Patients who died from intercurrent disease during the trial were censored at the date of death. Patients lost to follow-up were censored at the date of the last examination.

Correlations between clinical features and the expression of cytokeratins were evaluated statistically by chi-square tests. The overall survival curves were constructed according to Kaplan and Meier [15]. The Log-Rank test was used to assess differences between groups and the multivariate survival analysis was performed with Cox regression [16], a p-value < 0.05 was considered to be significant.

## Results

The series comprised 308 patients (240 men and 68 women) with a median age of 58 years (range 31–90). All tumours were classified post surgically according to the TNM system [12]. Patients were clinically evaluated in our routine follow-up for 3–10 years. Clinical and tumour details of the patients with oral squamous cell cancer are shown in Table 2.

On average 90 % of all cores could be evaluated for the expression of intermediate filaments.

Immunohistochemical examination showed that Ck 8/18, Ck 5/6, Ck 1, CK 10, Ck 14, Ck 19 and vimentin reactivity was confined homogeneously to the cytoplasma of neoplastic cells. The expression levels did not differ significantly for the vast majority of the cases between the two tumour cores representing one case, indicating a rather homogeneous expression. No correlation was found between Ck 1, Ck 5/6, Ck 10, Ck 14, vimentin and the survival prognosis.

**Figure 1:**
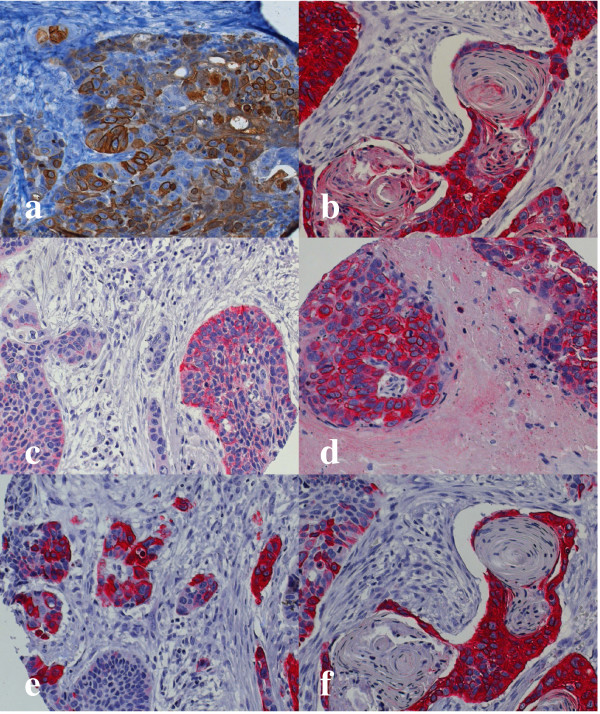
Immunohistochemical staining of oral squamous cell carcinoma with cytokeratin cytokeratin antibodies. a) cytokeratin 5/6 Antibody (magnification 20×) b) cytokeratin 19 antibody (magnification 20×) c) cytokeratin 8/18 antibody (Magnification 10×) d) cytokeratin 8/18 antibody (magnification 20×) e) cytokeratin 14 antibody (magnification 20×) d) cytokeratin 14 antibody (magnification 20×).

Detectable levels of Ck 8/18 (Ck 8/18 ≥ 1%) were found in 54 % (154/287) of the oral SCCs. Detectable levels of Ck 19 (Ck 19 ≥ 1%) were found in 37,5% (96/256) of the cases, high levels of Ck 19 expression (Ck 19 > 50%) were found in 4,2% (11/256) of the investigated tumour specimens (Table 3). The immunohistochemical staining is shown in Figure 1.

Ck 5/6 and Ck 14 were significantly correlated with the tumour size and lymph node status in the Chi-square Test (Table 4) and between each other (p < 0.01). No correlation was found between tumour size, tumour differentiation, lymph node status and the expression of Ck 8/18, Ck 1, Ck 10, Ck 19 and vimentin in Chi-square test.

**Figure 2:**
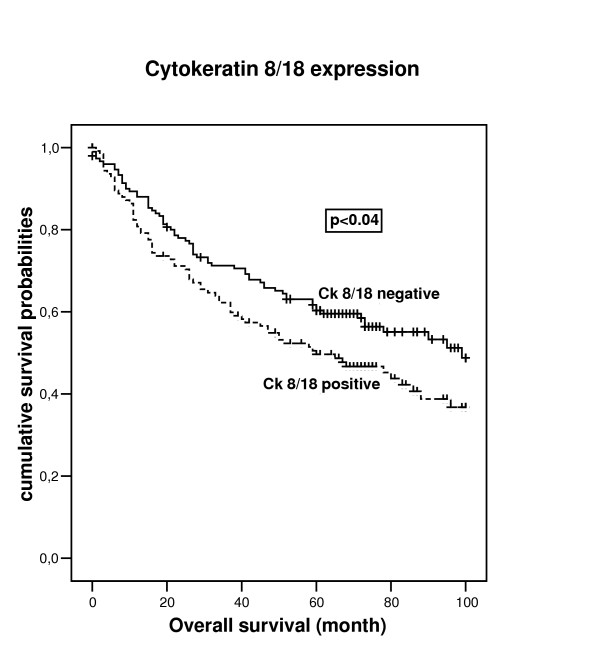
Overall survival of patient with oral squamous cell carcinoma in dependence on Cytokeratin 8–18 expression calculated by the Kaplan-Meier method.

**Figure 3:**
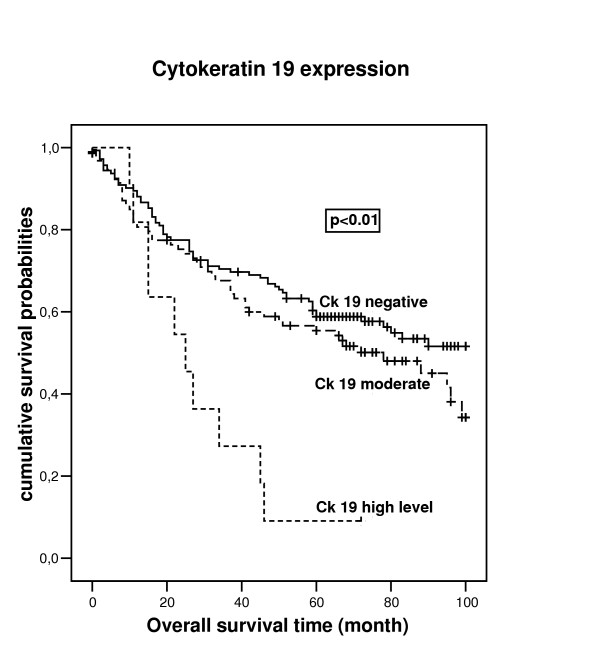
Overall survival of patient with oral squamous cell carcinoma in dependence on Cytokeratin 19 expression calculated by the Kaplan-Meier method.

Log-rank test of Ck 8/18 and Ck 19 expression showed a significantly decreased overall survival of carcinomas with a Ck 8/18 expression (Ck 8/18 ≥ 1% (n = 154), p < 0.04, Fig. 2) and a significant decreased overall survival associated with increased expression level of CK 19 (CK 19< 1% (n = 149), Ck 19 = 1–49% (n = 96), Ck 19 >50% (n = 11), p < 0.01, Fig. 3)

**Figure 4:**
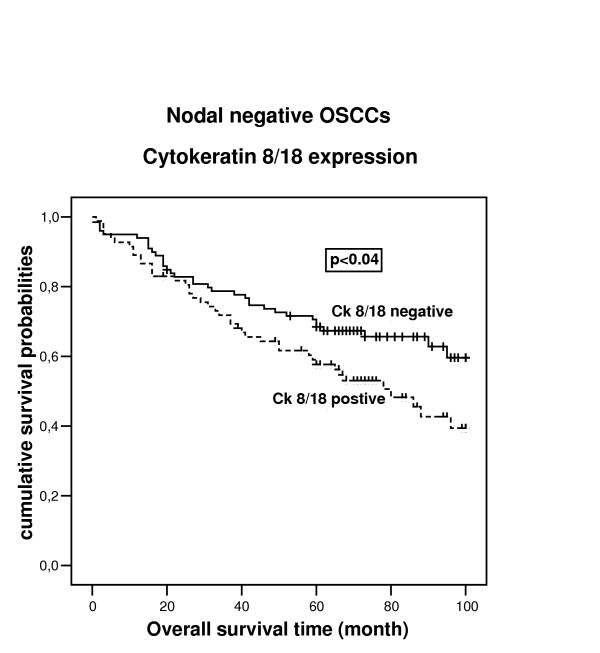
Overall survival of patient with nodal negative oral squamous cell carcinoma in dependence on Cytokeratin 8–18 expression calculated by the Kaplan-Meier method.

In the subgroup of node negative oral squamous cell cancer the expression of Ck 8/18 and the high expression of Ck 19 were also correlated with a poor overall survival (Ck 8/18, p < 0,04, Fig. 4) (Ck 19, p < 0.01). No influence of vimentin coexpression on the overall survival prognosis of Ck 8/18 and Ck 19 positive carcinomas was observed.

All carcinomas with an expression of Ck 8/18 and Ck 19 were reviewed and re-evaluated on whole sections. This was done in order to exclude the possibility of a misinterpretation and misclassification of dedifferentiated salivary gland carcinomas as poorly differentiated SCC's. However, in all these tumours the diagnosis of a primary salivary gland carcinoma could be ruled out.

In multivariate Cox analysis we compared the overall survival according to clinically established prognostic factors tumour size and nodal status with the expression of Ck 8/18, Ck 5/6, Ck 1, CK 10, Ck 14 and Ck 19 (Table 5). Three parameters as nodal status, tumour size and Ck 8/18 expression were indicated by Cox regression as independent predictors of survival prognosis.

## Discussion

Intermediate filaments are central components of the intracellular skeleton. The expression of distinct intermediate filament proteins is tissue-specific and almost highly conserved during carcinogenesis. Intermediate filaments are interpreted as mere bystanders and markers of distinct intracellular, cell-specific regulation mechanisms. Several studies associated changes in intermediate filament expression with an altered cellular behaviour [17,18].

The expression of high molecular weight cytokeratins, especially Ck 5 is a hallmark of squamous epithelium and is predominantly seen in the basal layers of stratified epithelium [2,19]. This cell layer is regarded as the anatomical localization of tissue specific stem/progenitor cells. Stem cells of stratified epithelium have been repeatedly described as the major cellular targets for cancer causing mutations and therefore might give in a long term rise to the development of SCC's. In the context of the existing literature it seems logical that SCC's retain and are phenotypically characterized by the expression of Ck 5. Adult healthy non-keratinizing stratified squamous epithelium of the upper aerodigestive tract express Ck 19 in basal layers but normally do not express Ck 8/18 [19]. Typical glandular cytokeratin Ck 8/18 are normally not expressed in SCC's [2,4]. On the other hand, Ck 8 and 18 is expressed in fetal buccal mucosa and tongue epithelium till 27 weeks of gestation [20].

Our study shows in a series of more than 300, uniformly treated, primary SCC's of the oral cavity that the expression of glandular cytokeratins such as Ck 8, 18 and 19, pinpoints a significant subgroup of SCC's. These carcinomas were associated with a poor prognosis, independently of the primary, preoperative lymph node status. It is important to stress that this held also true in multivariate analysis. This might be surprising on a first glance, since recent other clinico-pathological studies in SCC's of the oral cavity, especially with the focus on Ck 19 revealed inhomogeneous results [21-24]. This might on the one hand be due to significantly lower numbers of investigated tumour samples, and on the other hand caused by different techniques for the determination for the expression of cytokeratins 8/18 and 19 on the RNA and protein level. However, similar results to our findings have also been reported for skin carcinomas. Nevertheless, recent cell culture and studies with chemical carcinogens strongly support our results and revealed an important role of CK 8/18 in various steps of the pathogenesis and progression of SCC's. A spontaneous induction of Ck's 8 and 18 expression could be shown in SV40T-immortalized buccal mucosa cells, in tobacco induced leukoplakias and after introduction of v-H-Ras in epidermal mouse keratinocytes [25]. The transfection of buccal mucosa cells with a vector for Ck 8/18 resulted additionally in a significantly altered cellular morphology and increased cell motility – features associated with or being prerequisites for invasive tumour behaviour [10]. This was in line with previous experiments in melanoma cells [26]. The involvement of Ck 8/18 in SCC progression could further be demonstrated in chemically induced SCC's in the mouse skin. Whereas SCC's in mouse epidermis were Ck 8/18 positive, non-invasive papillomas did not show this feature. However, the underlying disturbed intra- and extra cellular regulation mechanisms have been characterized not in detail yet. A central role for TGF-alpha via the Epidermal Growth Factor Receptor (EGFR) has been postulated [27,28]. In contrast, the induction of Ck 8/18 expression in a similar experimental setting has also been described for TGF-alpha deficient mice, with putative involvement of H-Ras [29]. In consequence, multiple pathways in the regulation of Ck 8/18 are conceivably existing.

In general, cytokeratin expression patterns are highly-conserved. The above mentioned data from the literature showed that any change in the intermediate filament expression pattern reflects or underlies changes in substantial cellular properties. Strong homologies could also be shown in other carcinomas. In primary breast cancer, this could be demonstrated with different approaches. Down-regulated expression of cytokeratin Ck 8/18 was associated with increased metastatic properties and poor prognosis in breast cancer [30]. The expression of Ck 5 in a small subset of invasive breast cancers was in contrast associated with a poor prognosis [31].

Interestingly, the expression of vimentin, alone or in combination with Ck 8/18 and 19, was not associated with poor prognosis or higher tumour stages. A multitude of studies gave evidence that the expression of vimentin in carcinomas has to be interpreted as a sign of an epithelial-mesenchymal transition, associated with high tumour aggressiveness [9,32]. This association remains questionable, since the expression of vimentin has already been described in non-invasive premalignant lesions of squamous epithelium and cultured keratinocytes.

Finally, from a clinical point of view, our results show that the determination of the Cytokeratin 8–18 and 19-expression in SCC of the oral cavity harbours a significant prognostic potential. Our results seem to define subgroups of high-risk patients who might benefit from more intensive therapy or new therapeutic strategies like Cytokeratin 19 antibodies [33]. For human ovarian adenocarcinoma cells it could be shown that cytokeratins 18 could sensitize these cells to cisplatin [34]. These results demonstrate that modulating the expression of an intermediate filament protein results in sensitization to chemotherapeutic drugs. The subgroup of ck 8–18 positive squamous cell carcinoma may have a benefit by chemotherapy with cisplatin. Further investigations are necessary to determine the clinical impact of these hypotheses.

## Conclusion

In conclusion, our results in a series of 308 primary, previously untreated SCC's of the oral cavity showed an important prognostic impact for the expression of glandular, simple cytokeratins in SCC's. Further studies have to unravel the underlying regulatory changes in order to allow the definition of specific therapies of these high risk cancer patients.

## Competing interests

The author(s) declare that they have no competing interests.

## Authors' contributions

TF: Project planning, data analysis and writing of the manuscript

RW: Project planning and critical appraisal of the manuscript

JP: Data analysis and critical appraisal of the manuscript

BB: Project planning, critical appraisal of the manuscript

UJ: Critical appraisal of the manuscript

PM: Data analysis and critical appraisal of the manuscript

DW: Project planning and critical appraisal of the manuscript

HB: Project planning, data analysis and writing of the manuscript

## Pre-publication history

The pre-publication history for this paper can be accessed here:



## Figures and Tables

**Table 1:** Antibodies, company, clone, pre-treatment and dilution of used antibodies.

**Antibody**	**Company**	**Clone**	**Pre-treatment**	**Dilution**
Ck 5/6	DAKO, Glostrup, Denmark	D5/16B4	Autoclave 10 min	1 : 80
Ck 8/18	Dianova, Hamburg, Germany	K8.8/DC10	Autoclave 10 min, citrate buffer	1 : 40
Ck 1	Novocastra, Newcastle, UK	34βB4	Steamer 30 min, citrate buffer	1 : 150
Ck 10	DAKO, Glostrup, Denmark	DE-K10	Steamer 30 min, citrate buffer	1 : 400
Ck 14	Dianova Hamburg, Germany	LL002	Steamer 30 min, citrate buffer	1 : 50
Ck 19	Quartett GmbH, Berlin, Germany	KS19.1	Steamer 30 min, citrate buffer	1 : 80
Vimentin	Ventana Inc., Tucson, USA	V9	Steamer 30 min, citrate buffer	1 : 1000

**Table 2:** Tumour patient cases in this study

Age (mean)	58 years (range 31 – 90 years)
Sex	240 men, 68 women
Follow-up (mean)	64 months (range 4 – 181 months)
T stadium	135 T1 tumours
	125 T2 tumours
	15 T3 tumours
	33 T2 tumours
N stadium	212 N0 lymph node negative
	57 N1 lymph node positive carcinoma
	39 N2 lymph node positive carcinoma
Grading	54 well differentiated tumours (G1)
	214 moderately differentiated tumours (G2)
	40 weakly differentiated tumours (G3)

**Table 3:** Expression profile of cytokeratins

Antibody	No Expression	postive Expression
CK 5/6	123	169
CK8/18	133	154
CK 1	147	121
CK 10	177	91
CK14	159	107
Ck 19	149	107
Vimentin	237	24

**Table 4:** Chi-square Test of Ck 5–6 and Ck 14

Prognosticator	Node status P value	Tumour size P value
Ck 5–6	0.045	0.026
Ck 14	0.021	0.037

**Table 5:** Multivariate analysis of the time of survival in relation to the Cytokeratins expression and other prognostic factors

Prognosticator	P value^a^	RR^b^	Confidance interval^c^
Tumor size	*0.036	1.239	1.0–1.5
Nodal status	*0.000	1.866	1.4–2.4
CK5/6	0.350	1.167	0.8–1.6
CK1	0.931	0.985	0.7–1.4
CK10	0.852	0.968	0.7–1.4
CK19	0.227	1.163	0.9–1.5
CK14	0.470	0.872	0.6–1.3
CK8/18	*0.024	1.487	1.1–2.1

^a ^The partially nonparametric regression model of Cox (1972) was used to evaluate the predictive power of vatious combinations of prognosticators in a multivariate manner.

^b ^RR, relative risk

^c ^95% confidence interval
